# Text-in-Image Enhanced Self-Supervised Alignment Model for Aspect-Based Multimodal Sentiment Analysis on Social Media

**DOI:** 10.3390/s25082553

**Published:** 2025-04-17

**Authors:** Xuefeng Zhao, Yuxiang Wang, Zhaoman Zhong

**Affiliations:** School of Computer Engineering, Jiangsu Ocean University, Lianyungang 222005, China; 2022210911@jou.edu.cn (Y.W.); zhongzhaoman@163.com (Z.Z.)

**Keywords:** social media, aspect-based multimodal sentiment analysis, optical character recognition, modal alignment, self-supervised learning

## Abstract

The rapid development of social media has driven the need for opinion mining and sentiment analysis based on multimodal samples. As a fine-grained task within multimodal sentiment analysis, aspect-based multimodal sentiment analysis (ABMSA) enables the accurate and efficient determination of sentiment polarity for aspect-level targets. However, traditional ABMSA methods often perform suboptimally on social media samples, as the images in these samples typically contain embedded text that conventional models overlook. Such text influences sentiment judgment. To address this issue, we propose a text-in-image enhanced self-supervised alignment model (TESAM) that accounts for multimodal information more comprehensively. Specifically, we employed Optical Character Recognition technology to extract embedded text from images and, based on the principle that text-in-image is an integral part of the visual modality, fused it with visual features to obtain more comprehensive image representations. Additionally, we incorporate aspect words to guide the model in disregarding irrelevant semantic features, thereby reducing noise interference. Furthermore, to mitigate the semantic gap between modalities, we propose pre-training the feature extraction module with self-supervised alignment. During this pre-training stage, unimodal semantic embeddings from both modalities are aligned by calculating errors using Euclidean distance and cosine similarity. Experimental results demonstrate that TESAM achieved remarkable performances on three ABMSA benchmarks. These results validate the rationale and effectiveness of our proposed improvements.

## 1. Introduction

With the advancement of the Internet, an increasing number of individuals are inclined to express their opinions on social media platforms. Most of these opinions are conveyed through multimodal forms, combining text and images, which encapsulate rich sentiment polarity information directed at various aspects. To capture such nuanced information, the field of aspect-based multimodal sentiment analysis (ABMSA) has emerged.

**Figure 1:**
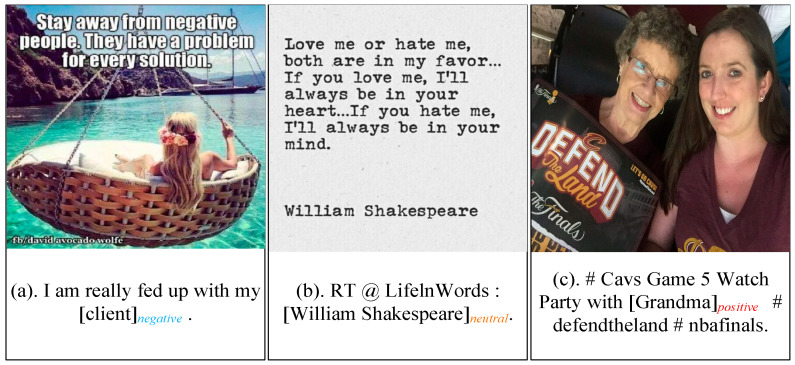
Example of the multimodal data. Within square brackets are aspect-level targets with sentiment labels appended to the lower right corner of the brackets, distinguished by different colors.

ABMSA is a subfield of multimodal sentiment analysis (MSA). By integrating information from multiple modalities, MSA offers more accurate extraction of sentiment features compared to single-modal methods. For social media platforms, MSA primarily focuses on text and images. The content in text-image pairs of social media exhibits rich interactions. First, there is a correspondence between the text and the image. For instance, in Figure 1a, the “client” mentioned in the text corresponds to the “negative people” in the embedded text in the image, while the reclining girl in the image suggests that “I” am trying to calm my emotions. Second, there is often a complementary relationship between modalities. For example, in Figure 1c, the text alone suggests that the sentimental polarity of “grandma” is likely not negative, but when supplemented with the smiling figure in the image, there is sufficient reason to determine her polarity as positive. Hence, by modeling the interactions between different modalities, MSA can effectively capture sentiment components that may be difficult to infer from a single modality alone, thereby providing more precise sentiment analysis for aspect-based targets.

Nevertheless, accurately capturing cross-modal interactions of the same aspect across different modalities remains one of the key challenges for ABMSA. Currently, researchers [1,2,3,4,5] tend to extract image and text features separately and then apply attention mechanisms to fuse them. However, images on social media often contain embedded text (text-in-image), which can influence how users interpret the overall content. For example, the embedded text in Figure 1a conveys a sentiment polarity that is entirely opposite to the overall sentiment of the image, while the image modality in Figure 1b consists solely of text, offering little visual information for reference. As a result, disregarding text-in-images may lead to errors in sentiment analysis. Moreover, the model’s performance is also influenced by the differences in modal representations, which may introduce additional noise during the feature fusion phase. Some existing methods [6,7,8,9] mitigate such noise by converting images into textual representations (e.g., image descriptions, object descriptions, or emotion annotations). However, these approaches require additional image understanding models as support, leading to increased training and inference costs.

To address these challenges, we propose a text-in-image enhanced self-supervised alignment model (TESAM). Specifically, we first design a novel image feature extraction module that simultaneously captures both visual features and text-in-image. Since text-in-image should be regarded as part of the image modality, we have customized the image feature extractor to fuse visual features with these texts, and use aspect words to guide the model’s focus on the image modality. This approach maps visual image and text-in-image to the same embedding space, enhancing the original visual representation. Next, we introduce a bidirectional interaction module to capture the bidirectional interaction between enhanced image representation (EIR) and the extracted textual features, allowing us to identify how the same aspect is expressed across different modalities and extract the associated sentiment components. Before model training, we propose using multiple distance metrics to pre-train the feature extraction module on the image caption dataset using self-supervised learning. This approach aims to narrow the semantic gap between modalities and reduce the introduction of additional noise. Finally, through a classification module, the extracted sentiment features are categorized into distinct sentiment polarity classes for output.

In summary, the main contributions of this paper can be outlined as follows:

(1) We propose a text-in-image enhanced self-supervised alignment model (TESAM) for social media sentiment analysis. By incorporating text-in-image, TESAM enables more comprehensive sentiment-level analysis of multimodal content compared to existing models, reducing misjudgments caused by information loss.
(2) We design a text-in-image-enhanced image feature extraction module. This module simultaneously extracts visual features and embedded text, employing an early fusion strategy with an aspect-guided attention mechanism to integrate both modalities, thereby obtaining more comprehensive image representations.
(3) We introduce a self-supervised pre-training paradigm using an image-caption dataset to align the model’s feature extraction modules before formal training. By learning from highly correlated image-text pairs, this approach mitigates modal gaps, reduces additional noise during subsequent feature fusion, and enhances model performance.
(4) We conduct extensive and detailed experiments on three ABMSA benchmarks, demonstrating that the proposed method achieves a performance exceeding that of traditional approaches.

## 2. Related Works

In this section, we will provide a concise overview of prior contributions, encompassing the fields of ABMSA and optical character recognition (OCR).

### 2.1. Aspect-Based Multimodal Sentiment Analysis

Aspect-based multimodal sentiment analysis usually detects an aspect’s sentiment by combining expressions across different modalities, such as textual, image, video, and auditory information [10]. This approach enables a more comprehensive analysis of sentiments, expressions, and context, particularly in applications where sentiments are conveyed through diverse channels [11,12].

For social media, the primary modalities are image and text, which contain rich aspect-level sentiment polarities that can be extracted and analyzed. Xu et al. [13] are pioneers in advancing the ABMSA task, proposing the Multi-Interactive Memory Network (MIMN). This model combines attention and gating modules to learn the interaction effects between multimodal data and the self-influence within unimodal data, enabling aspect-level sentiment analysis. However, the fusion method used in MIMN is relatively basic, and the extraction of sentiment features is not comprehensive enough. Therefore, with the rise of transformer-based models [14], Yu et al. [1] introduced an additional image branch into BERT (Bidirectional Encoder Representations from Transformers), enabling aspect words to query images to support text sentiment analysis. They proposed the Target-Oriented Multimodal BERT (TomBERT), characterized by the integration of cross-modal attention to generate aspect-sensitive representations. However, TomBERT still focused primarily on text and did not fully exploit image features. Subsequently, Yu et al. [4] advanced their work by proposing the Hierarchical Interactive Multimodal Transformer (HIMT). HIMT utilized Faster R-CNN (Region- Convolutional Neural Networks) [15] to enrich image representations and designed an auxiliary reconstruction module to mitigate the semantic gap between modalities. Nevertheless, these methods overlook the influence of text-in-image, which is widely distributed across social media. Such text is challenging for image feature extractors to capture, but can provide critical insights for sentiment analysis.

Additionally, other efforts to bridge the gap include innovative approaches such as Khan et al.’s method [8], which employed self-supervised learning to transform image semantics into textual descriptions using image understanding models. Zhao et al. [5] proposed generating adjective–noun pairs (ANPs) to aid in modality alignment. Li et al. [6] enriched image modality representation by converting images into textual forms, including image descriptions, facial expressions, and OCR-extracted text. Though these methods effectively align images with the text modality, they rely on additional image understanding models, which can increase training and inference costs. Moreover, their handling of text-in-image remains limited. For instance, Li et al. [6] simply concatenate textual descriptions of images without leveraging aspect words to guide the alignment process.

To address these limitations, we introduce text-in-image with inserted aspect words to enable a more comprehensive sentiment analysis of multimodal content. We also propose leveraging additional highly correlated image-text pairs to align different modalities, rather than focusing on the low image-text correlation in the ABMSA task datasets.

### 2.2. Optical Character Recognition

Optical character recognition refers to the technology used for detecting and converting printed or handwritten text in images into machine-readable text.

Traditional OCR methods are generally based on template matching, wherein the similarity between an image region and predefined templates is calculated to select the most similar category, thereby identifying potential characters in the image. These methods are limited to specific fonts and contexts, suffering from poor generalization capabilities, and thus are not widely applicable. More advanced OCR approaches leverage deep learning techniques and typically involve two stages: detection and recognition. In the detection phase, some studies have developed highly accurate algorithms based on Regions with CNN (RCNN) [16] and its improved model, Faster R-CNN [15], while others have applied single-step detection models like SSD (Single Shot MultiBox Detector) [17] or YOLO (You Only Look Once) [18] to achieve faster detection speeds. Once the character locations are identified, classification is typically handled by visual recognition methods based on CNN or sequence recognition methods based on RNN. These methods achieve higher accuracy by autonomously learning the necessary features, making them more adaptable to various scenarios. However, CNNs struggle to recognize character sequences in images, while RNNs cannot be trained end-to-end. To address these limitations, Shi et al. [19] proposed the Convolutional Recurrent Neural Network (CRNN) architecture, which combines CNN and RNN (Recurrent Neural Network). This approach preserves the end-to-end training paradigm while enabling sequence recognition, demonstrating state-of-the-art performance in comparative experiments.

In the context of multimodal sentiment analysis, OCR plays an essential role by extracting embedded text from images that can significantly influence interpretation. Incorporating text-in-image in multimodal sentiment analysis allows for a fuller understanding of both visual and textual elements, providing a more comprehensive assessment of the content’s sentiment.

## 3. Methods

### 3.1. Task Definition

Given a set $S=\{x_{1},x_{2},\ldots ,x_{i},\ldots ,x_{Z}\}$ of multimodal samples, in which $x_{i}=\{T_{i},I_{i},A_{i}\}$ represents one pair of text-image samples with a tagged aspect, *T_i_* is a sequence of word tokens $\left(w_{1},w_{2},\ldots w_{n}\right)$, *I_i_* is an image tensor of shape (3,224,224), and *A_i_* is the aspect word(s) in *T_i_*. Further, $x_{i}$ is associated with a label $y_{i}\in \{1,2,\ldots ,l\}$, where *l* is the number of sentiment classes. The value of *y_i_* represents the sentiment polarity of the aspect word(s) *A_i_*, from 1 (negative) to *l* (positive). Our goal is to use S to train a model that can correctly predict *y* for each input of x.

### 3.2. Overview of Our Proposed Model

**Figure 2:**
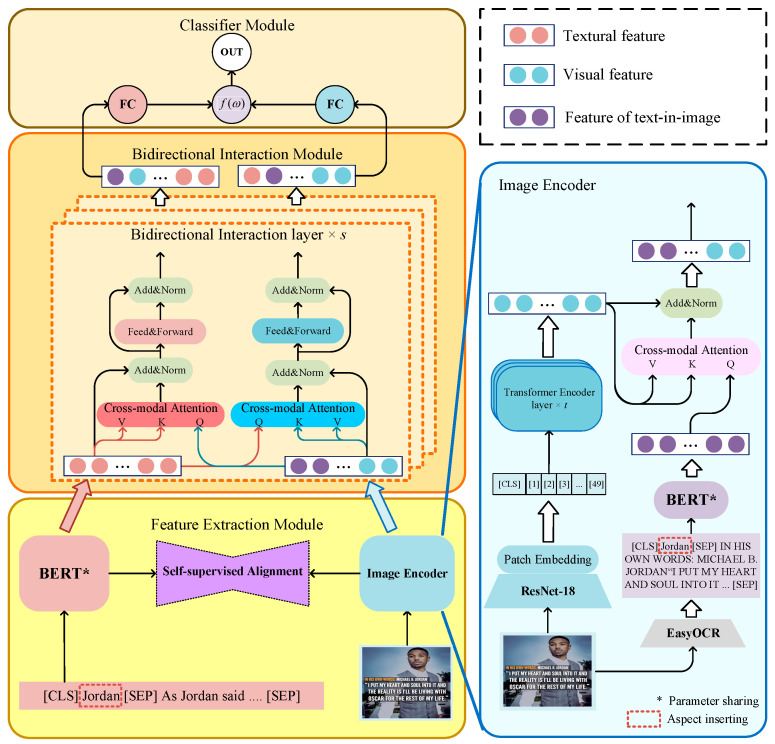
Structure of the proposed model. The thin arrows indicate the model’s inference path, while the thick arrows denote visualization of the output of the previous component.

Figure 2 illustrates the overall architecture of TESAM. Considering that the extraction of sentiment components is a process that progresses from low-level features to high-level features and from unimodal to multimodal, TESAM is divided into three modules: (1) feature extraction module: In this module, we innovatively incorporate the features from text-in-images and fuse them with the pure visual features extracted through convolutional neural networks (CNN). This process encodes both types of features into the same embedding space, resulting in an enhanced image representation (EIR). For the textual modality, we utilize the advanced pre-trained language model BERT to extract semantic features, which serve as the foundation for multimodal analysis. (2) Bidirectional interaction module: This module is designed to capture the bidirectional interactions between EIR and textual representation, then identify how the same aspect is expressed across different modalities. By doing so, it enables more accurate extraction of sentiment components relevant to specific aspects in both image and text. (3) Classification module: After obtaining a comprehensive multimodal representation, the extracted sentiment features are passed through a classification module. This module distinguishes the sentiment polarities (e.g., negative, neutral, positive) and outputs the final sentiment classification based on the combined multimodal analysis.

### 3.3. Feature Extraction Module

We design a feature extraction module to extract preliminary sentimental representations of images and texts as the basis for sentiment analysis.

#### 3.3.1. BERT for Text Feature Extraction

To convert the text sample *T_i_* into textural feature $P_{i}^{T}\in {\mathbb{R}}^{N\times d}$ (*N* is the length of the input sequence, *d* is the dimension of the embeddings), we adopt BERT [14], a pre-trained masked language model (MLM) that has achieved state-of-the-art performance in many natural language processing (NLP) tasks, as the text feature extractor. As shown in Table 1, the input text fed into BERT is augmented with “[CLS]” and “[SEP]” tokens to mark the beginning and break or end of the sentence, respectively. For the ABMSA task, $A_{i}$ is inserted into the input, indicating the target of sentiment analysis.

BERT accepts a sequence of fixed-length tokens *T_i_* as input and is able to capture long-distance dependencies in the text through the multi-head self-attention mechanism. First, BERT converts *T_i_* into $E_{i}\in {\mathbb{R}}^{N\times d}$ through word embedding:

(1) $$E_{i}=\mathrm{Embedding}(T_{i})$$

Then, BERT_base_ stacks transformer encoder (TE) layers 12 times to obtain $P_{i}^{T}$:

(2) $$P_{i}^{T}={\mathrm{BERT}}_{base}(E_{i})={\mathrm{TE}}^{\left(12\right)}(E_{i}).$$

#### 3.3.2. Image Encoder for Enhanced Image Feature Extraction

ResNet [20] is a lightweight residual CNN that has achieved remarkable results on the ImageNet dataset. For the image modality, ResNet-18 is employed to extract pure visual feature $V_{i}\in {\mathbb{R}}^{d\times w\times h}$ from resized image sample $I_{i}\in {\mathbb{R}}^{3\times 224\times 224}$:

(3) $$V_{i}=W_{D}{\mathrm{ResNet}}_{18}(I_{i}),$$

where $W_{D}\in {\mathbb{R}}^{d\times c}$ is applied to map visual features with *c* channels into a *d*-dimensional embedding space. Compared to other deeper versions, ResNet-18 has fewer parameters while achieving comparable performance, making it suitable for preliminary visual feature extraction.

Subsequently, to map and encode the pure visual features into the same embedding space as text features, we first utilize patch embedding [21] and transformer encoder layers:

(4) $$V_{i}^{P}={\mathrm{TE}}^{\left(t\right)}([\mathrm{CLS},\mathrm{Flatten}(\mathrm{Conv}(V_{i}))]+P_{os}),$$

Here, Conv(·) represents the convolutional layer for dimension mapping, as the foundation for modality alignment. Flatten(·) reshapes the feature map generated by the convolutional layer into a sequence to concatenate with the [CLS] token. “CLS” is a randomly initialized classification token, while $P_{os}$ represents the randomly initialized positional encoding. The addition of the positional encoding enables the model to automatically learn and reconstruct the spatial relationships between image patches [21]. Additionally, *t* represents the number of stacked TE layers, the specific value of which will be discussed in detail in the Section 4.

In addition to pure visual features, the embedded text in images may contain information that is crucial for aspect-based sentiment analysis. Thus, text-in-image is often an essential component that cannot be overlooked. These texts may be presented in various fonts, colors, and orientations, making extraction challenging. Therefore, we chose to employ EasyOCR [22], an open-source text recognition engine developed by JaidedAI, to identify the text-in-image:

(5) $$V_{i}^{\prime }=["[CLS]",A_{i},"[SEP]",\mathrm{EasyOCR}(I_{i}),"[SEP]"],$$

where $V_{i}^{\prime }$ is a sequence of text, and “[·, ·]” denotes the concatenation operation. By inserting the aspect word $A_{i}$ into the text-in-image, the model’s attention is directed towards image regions associated with $A_{i}$, enabling the extraction of specific sentiment components while minimizing interference from irrelevant noise.

EasyOCR demonstrates state-of-the-art performance, enabling rapid localization and recognition of text-in-images, thereby supplementing the semantic features of the image modality. This enhancement provides more comprehensive contexts for ABMSA, improving classification accuracy and making it suitable for our targeted social media platforms.

Thus, for the text-in-image, we employ BERT to extract its semantic features, thereby enhancing the overall representation of the sample:

(6) $$V_{i}^{T}={\mathrm{BERT}}_{base}^{*}(V_{i}^{\prime }),$$

where “*” indicates that this BERT shares trainable parameters with BERT in Equation (1). By applying parameter sharing, we significantly reduce the number of trainable parameters in the model while ensuring the effective extraction of features, thereby lowering the costs associated with model training.

Next, we extended self-attention [14] to a multi-head cross-modal attention (MCA) mechanism. Specifically, in self-attention, the input is copied into queries, keys, and values. The query vector determines what content to focus on in the mechanism, while the key and value vectors represent the information of the queried objects. Therefore, by modifying the queries to vectors that come from visual modality, MCA is able to highlight sentimental relationships from embedded text to image:

(7) $${\mathrm{head}}_{j}=\mathrm{softmax}(\frac{[{\tilde{W}}_{Q}V_{i}^{T}]{\left[{\tilde{W}}_{K}V_{i}^{P}\right]}^{T}}{\sqrt{d/m}})[{\tilde{W}}_{V}V_{i}^{P}],$$

where ${\tilde{W}}_{\{Q_{j},K_{j},V_{j},M\}}$ are the weights of linear layers and ${\tilde{W}}_{\{Q_{j},K_{j}\}}\in {\mathbb{R}}^{(d/r)\times d}$, ${\tilde{W}}_{V_{j}}\in {\mathbb{R}}^{d\times d}$, ${\tilde{W}}_{M}\in {\mathbb{R}}^{m\times 1}$. In addition, *m* is the number of attention heads. This attention mechanism facilitates interaction between the two types of features to capture the full context of the image, including both visual and textual cues.

At last, we utilize skip connections and layer normalization (LN) to construct the image encoder (IE) and output EIR $P_{i}^{V}\in {\mathbb{R}}^{d\times w\times h}$:

(8) $$P_{V_{i}}=\mathrm{IE}(V_{i})=\mathrm{LN}({\bar{V}}_{i}+V_{i}^{P}).$$

### 3.4. Bidirectional Interaction Module

The bidirectional interaction (BI) module consists of stacked bidirectional interaction layers, which use cross-modal attention to capture the rich interaction between EIR and text feature, further extracting cross-modal sentimental feature, and ultimately obtaining high-level sentiment representation (HSR). Since the EIR and textual features reside in the same dimensional embedding space, we designed this module with a symmetrical structure, encompassing cross-modal fusion from image to text as well as from text to image.

#### 3.4.1. Image-to-Text Cross-Modal Fusion

To enable interaction from $P_{i}^{V}$ to $P_{i}^{T}$, similar to the cross-modal attention mechanism in the IE, we applied ${\mathrm{MCA}}_{V\to T}$ to highlight a sentimental relationship from image to text:

(9) $${\mathrm{MCA}}_{V\to T}(P_{i}^{V},P_{i}^{T})=[{\mathrm{head}}_{1},\ldots ,{\mathrm{head}}_{m}]{\hat{W}}_{M},$$

where ${\hat{W}}_{M}\in {\mathbb{R}}^{m\times 1}$ is the weight parameter, and the *j*-th head:

(10) $${\mathrm{head}}_{j}(P_{i}^{V},P_{i}^{T})=\mathrm{softmax}(\frac{[P_{i}^{V}{\hat{W}}_{Q_{j}}]{\left[P_{i}^{T}{\hat{W}}_{K_{j}}\right]}^{T}}{\sqrt{d/m}})[P_{i}^{T}{\hat{W}}_{V_{j}}],$$

where ${\hat{W}}_{\{Q_{j},K_{j},V_{j}\}}\in {\mathbb{R}}^{d\times (d/m)}$ are attention weight parameters to be trained. In our model, ${\mathrm{MCA}}_{V\to T}$ helps the model highlight the text related to the aspect in the image, thereby achieving the goal of feature fusion.

Next, we use FNN (Feedforward Neural Network) and layer normalization to further extract the fused features:

(11) $$F_{V\to T}(P_{i}^{V},P_{i}^{T})=\mathrm{LN}(\hat{L}+\mathrm{FNN}(\hat{L})),$$

where $\hat{L}=\mathrm{LN}({\mathrm{MCA}}_{V\to T}((P_{i}^{V},P_{i}^{T}))+P_{i}^{V}).$

This structure further facilitates the processing and refinement of the extracted features, enabling the model to effectively focus on the sentiment information in the text that is relevant to the image, thereby eliminating the interference of irrelevant components on the classification results.

#### 3.4.2. Text-to-Image Cross-Modal Fusion

Firstly, similar to ${\mathrm{MCA}}_{V\to T}$, we replace the query input of multi-head attention with image features enhanced by text-in-image to design ${\mathrm{MCA}}_{T\to V}$:

(12) $${\mathrm{MCA}}_{T\to V}(P_{i}^{V},P_{i}^{T})=[{\mathrm{head}}_{1},\ldots ,{\mathrm{head}}_{m}]W_{M}^{\prime },$$

(13) $${\mathrm{head}}_{j}(P_{i}^{V},P_{i}^{T})=\mathrm{softmax}(\frac{[P_{i}^{T}W_{Qj}^{\prime }]{\left[P_{i}^{V}W_{Kj}^{\prime }\right]}^{T}}{\sqrt{d/m}})[P_{i}^{V}W_{Vj}^{\prime }],$$

where $W_{\{Q_{j},K_{j},V_{j},M\}}^{\prime }$ are the weights of the linear layers. This highlights the parts of EIR that are most closely associated with the sentiment in text feature through correlation calculation.

Next, ${\mathrm{MCA}}_{T\to V}$ and FNN together constitute the sentiment encoder on the image side. Through $F_{T\to V}$, the EIR that has been encoded with sentiment in text after interaction becomes the hidden image representation with more prominent sentimental features:

(14) $$F_{T\to V}(P_{i}^{V},P_{i}^{T})=\mathrm{LN}(L^{\prime }+\mathrm{FNN}(L^{\prime })),$$

where $L^{\prime }=\mathrm{BN}({\mathrm{CA}}_{T\to V}(P_{i}^{V},P_{i}^{T})+P_{i}^{V}).$ $F_{T\to V}$ enhances the understanding of the sentimental content in images by incorporating textual information, thereby enriching the multimodal representation of sentiments in the high-level image feature.

Finally, the entire bidirectional interaction layer is stacked *s* times to fully facilitate multimodal interaction between text and image:

(15) $$(H_{i}^{V},H_{i}^{T})={\left(F_{V\to T}(P_{i}^{V},P_{i}^{T}),F_{T\to V}(P_{i}^{V},P_{i}^{T})\right)}^{\left(s\right)},$$

leading to the final sentiment representation $\left(H_{i}^{V},H_{i}^{T}\right)$. The impact of the value of *s* on the model’s performance will be discussed in the Section 4.

### 3.5. Classification Module

The final sentiment representation highly abstracted sentiment features and can be directly utilized for classification. Therefore, we employ fully connected layers to, respectively, map it to decision-level sentiment scores $D_{i}^{\left\{V,T\right\}}\in {\mathbb{R}}^{1\times l}$. To be specific, the hidden state of the [CLS] token $H_{i}^{\left\{V,T\}[CLS\right]}\in {\mathbb{R}}^{1\times d}$ is defined as the holistic representation of the two sequences for classification, so we pick them as the sentiment feature representations for the entire sample. Then, $H_{i}^{\left\{V,T\}[CLS\right]}$ is passed through FNN to derive $D_{i}^{\left\{V,T\right\}}$:

(16) $$D_{i}^{V}=\mathrm{FNN}(H_{i}^{V[CLS]}),$$

(17) $$D_{i}^{T}=\mathrm{FNN}(H_{i}^{T[CLS]}).$$

Subsequently, a weighted average function combined with softmax normalization is applied to determine the prediction class $O_{i}\in {\mathbb{R}}^{1}$ for each sentiment category:

(18) $$O_{i}=\mathrm{argmax}(\mathrm{softmax}(D_{i}^{V}\omega +D_{i}^{T}(1-\omega ))),$$

where $\omega \in {\mathbb{R}}^{l}$ is a weight parameter, and argmax(⋅) selects the index of the maximum value in a vector as its output.

### 3.6. Self-Supervised Alignment

In multimodal learning, the alignment of features between image and text is crucial. Absence of alignment may increase the difficulty of model training, as there are significant differences in the representations provided by their respective feature extractors in the feature space. Thus, we propose performing self-supervised learning to effectively align image and text representations before the formal training process. This helps to reduce the semantic gap between the two modalities, minimizes the noise interference caused by feature fusion, and ultimately improves the model’s performance. During self-supervised alignment, we use an additional image captioning dataset instead of the task-specific ABMSA dataset. This is because the ABMSA dataset often contains a significant amount of noise and interference, with some instances where the image and text are irrelevant. In contrast, image captioning datasets are designed from the outset with highly relevant image-text pairs. This high semantic consistency between image and text is able to build the foundation for effective modality alignment.

To perform self-supervised alignment, we divide the model training into two stages. In the pre-training stage, we train the feature extraction module of TESAM using an image captioning dataset. First, we input the image and its corresponding caption into the feature extraction module to obtain the representations $P_{i}^{\left\{T,V\right\}}$ for each modality:

(19) $$P_{i}^{T}={\mathrm{BERT}}_{base}(E_{i}),$$

(20) $$P_{i}^{V}={\mathrm{IE}}_{-OCR}(V_{i}).$$

In Equation (20), ${\mathrm{IE}}_{-OCR}$ refers to the removal of the text-in-image-related components in the IE, leaving only the components responsible for pure visual feature extraction. Specifically, EasyOCR and BERT* in the IE module are removed (for images in the image caption dataset almost never contain text), and the output of TE is used as the query sequence for the cross-modal attention, reducing it to self-attention. Since the aligned features from ResNet+TE and BERT are located in the same embedding space and can generate correlated feature representations for semantically related content, the cross-modal attention mechanism is less likely to be affected. Moreover, BERT* in the IE module and the BERT used for textual feature extraction share the same weight parameters, allowing them to benefit from the advantages of self-supervised alignment. Thus, in the next step, we constructed the loss function using mean squared error (MSE) and cosine similarity (CosSim) for self-supervised alignment:

(21) $$\begin{array}{l} L_{sa}=\frac{1}{2}(\mathrm{MSE}(P^{T[CLS]},P^{V[CLS]})+\mathrm{CosSim}(P^{T[CLS]},P^{V[CLS]}))\\ =\frac{1}{2\left|S\right|}\sum_{i=1}^{S}\left[{\left(P_{i}^{T[CLS]}-P_{i}^{V[CLS]}\right)}^{2}+(1-\frac{P_{i}^{T[CLS]}\cdot P_{i}^{V[CLS]}}{\left\|P_{i}^{T[CLS]}\right\|\times \left\|P_{i}^{V[CLS]}\right\|})\right]. \end{array}$$

This design aims to comprehensively account for both the Euclidean distance and the angular relationship between modality representations in the embedding space. By doing so, it ensures that the feature extractor produces tightly coupled and consistent multimodal representations for semantically similar image-text pairs.

### 3.7. Loss Function

After employing self-supervised learning for modal alignment, in the training stage, we use cross-entropy loss to train the whole network. Cross-entropy loss directly measures the discrepancy between the category distribution of the model’s output and the distribution of true labels:

(22) $$L(y_{i},O_{i})=-\log p(y_{i}|O_{i}),$$

where $y_{i}$ indicates the true label of the input multimodal data.

### 3.8. Training Procedure

The pseudocode in Algorithm 1 outlines the training procedure of TESAM. First, the feature extraction module undergoes self-supervised pre-training on the COCO-Captions dataset to align its image and text feature outputs. Then, the aligned feature extraction module parameters are transferred to TESAM, which is fine-tuned on the ABMSA task dataset. However, at this stage, the bidirectional interaction module and classification module remain untrained, with randomly initialized parameters. Therefore, during the initial fine-tuning phase, the feature extraction module’s parameters are frozen while the other parameters are warmed up. Finally, TESAM undergoes full fine-tuning to achieve multimodal sentiment analysis. The learning rates follow a decay strategy, the specific update rules are listed in Section 4.2.
**Algorithm 1** Training procedure of TESAM**Input:** Dataset for alignment $D_{A}$, task-specific dataset $D_{T}$, Image Encoder’s parameters $\theta_{\mathrm{img}}$, Text Encoder’s parameters $\theta_{\mathrm{text}}$, TESAM’s parameters $\theta_{T}=\{\theta_{\mathrm{img}},\theta_{\mathrm{text}},\theta_{\mathrm{other}}\}$, training epochs $E_{1}$ and $E_{2}$, learning rates $\eta_{1}$ and $\eta_{2}$ **# Phase 1:** Self-supervised Alignment 1:  **for** *epoch* **in** $1,\ldots ,E_{1}$ **do** 2:    **for** $\left(T_{i},I_{i}\right)$ **in** $D_{A}$ **do** 3:     $t={\mathrm{BERT}}_{base}(T_{i};\theta_{\mathrm{text}})$ # extract text feature 4:     $v={\mathrm{IE}}_{-OCR}(I_{i};\theta_{\mathrm{img}})$ # extract visual feature 5:     $L_{\mathrm{align}}=\frac{1}{2}(\mathrm{MSE}(t^{\left[CLS\right]},v^{\left[CLS\right]})+\mathrm{CosSim}(t^{\left[CLS\right]},v^{\left[CLS\right]}))$ # alignment loss 6:     $\theta_{\mathrm{text}}\leftarrow \theta_{\mathrm{text}}-\eta_{1}\nabla_{\theta_{\mathrm{text}}}L_{\mathrm{align}}$ # update parameters 7:     $\theta_{\mathrm{img}}\leftarrow \theta_{\mathrm{img}}-\eta_{1}\nabla_{\theta_{\mathrm{img}}}L_{\mathrm{align}}$ # update parameters 8:     $\eta_{1}\leftarrow \mathrm{update}(\eta_{1})$ # update learning rate 9:    **end for** 10: **end for** # **Phase 2:** Fine-tuning on task-specific dataset 11: $\theta_{T}\leftarrow \{\theta_{\mathrm{img}},\theta_{\mathrm{text}}\}$ # transfer aligned encoder parameters to TESAM 12: **for** *epoch* **in** $1,\ldots ,E_{2}$ **do** 13:    **for** $\left(T_{i},I_{i},A_{i},y_{i}\right)$ **in** $D_{T}$ **do** 14:     $O_{i}=\mathrm{TESAM}(T_{i},I_{i},A_{i};\theta_{\mathrm{img}},\theta_{\mathrm{text}},\theta_{\mathrm{other}})$ # predict sentiment label 15:     $L_{c}=-\log p(y_{i}|O_{i})$ # classification loss 16:     **if** ($epoch<\left\lceil E_{2}/4\right\rceil$) **then** 17:       $\theta_{\mathrm{other}}\leftarrow \theta_{\mathrm{other}}-\eta_{2}\nabla_{\theta_{\mathrm{other}}}L_{c}$ # warm up other parameters in TESAM 18:     **else** 19:       $\theta_{T}\leftarrow \theta_{T}-\eta_{2}\nabla_{\theta_{T}}L_{c}$ # fine-tune the whole model 20:     **end if** 21:     $\eta_{2}\leftarrow \mathrm{update}(\eta_{2})$ # update learning rate 22:    **end for** 23: **end for**

## 4. Experiment

### 4.1. Datasets

#### 4.1.1. TWITTER Datasets and Multi-ZOL Dataset for ABMSA Task

To comprehensively demonstrate the effectiveness of our proposed method, we conducted experiments using a finer-grained ABMSA task. So, we choose 3 benchmark datasets—TWITTER-15, TWITTER-17 [1], and Multi-ZOL [13]—for our experiments.

The TWITTER datasets comprise tweets gathered from 2015 to 2017. Each data instance in these datasets corresponds to one or more aspect-level texts (targets), with each target assigned one of three sentiment polarities {negative, neutral, positive}.

The Multi-ZOL dataset is used to verify the generalization ability of TESAM in different scenarios. It is collected from the Chinese technology forum ZOL.com, specifically from mobile phone reviews. Each sample includes multiple evaluation aspects, which are cost–performance ratio, performance configuration, battery life, appearance and feel, camera effect, and screen effect. The sentiment tendency for each aspect is represented by a sentiment score ranging from 1 (negative) to 10 (positive).

Details of these datasets are listed in Table 2 and Table 3, including the number of samples in each classification category for the subsets of each dataset, as well as the proportion of samples with optical characters in their images. It can be observed that more than 50% of the sample images in the TWITTER-15 dataset contain text, while this proportion exceeds 60% in the TWITTER-17 and Multi-ZOL datasets. This demonstrates the rationality of our improvement in enhancing the model’s visual representations through the introduction of text-in-image.

#### 4.1.2. COCO-Captions for Self-Supervised Alignment

The COCO-Captions dataset [23] is an extension of the Microsoft Common Objects in Context (COCO) dataset, designed for image captioning tasks. It includes over 120,000 images with around 600,000 captions, making it one of the largest and most widely used datasets for image-to-text research. Each image in the dataset is associated with multiple captions (typically five), providing diverse linguistic descriptions for the same visual content. Thus, COCO-Captions is well-suited as a dataset for self-supervised modal alignment during the pre-training stage of TESAM.

### 4.2. Parameter Settings

In our experiments, for the text modality, the maximum number of text tokens *N* is set to 50 to reduce memory use and match the number of image patches. The number of attention heads *m* is 12, and the output text embedding dimension *d* is 768. For the image modality, the original images are resized to 256 × 256 and then randomly cropped to 224 × 224 before being normalized and input to the model. The model is trained using the Adam [24] optimizer with parameters: *betas* = (0.9, 0.999), *eps* = 1 × 10^−8^, *weight_decay* = 0 from experience. In the self-supervised alignment stage, the learning rate is set to 3 × 10^−5^, with a 50% decrease every epoch. The batch size is 64, and the feature extraction module is pre-trained for a total of 6 epochs. For the Chinese Multi-ZOL dataset, we employed a translated Chinese version of the COCO-Captions dataset for self-supervised alignment. In the training stage, the initial learning rate of the model is set to 5 × 10^−5^, and it is decreased by 70% every 2 epochs. Dropout is set to 0.01, and the batch size is set to 32. The model is implemented using PyTorch 2.0.1 and trained on an NVIDIA RTX 4080 GPU (manufactured in Shenzhen, China). For 8 epochs on each dataset. All random seeds are set to 42.

### 4.3. Baselines

We choose the following baselines for comparison.

CLIP [20]: Maps images and text into shared embedding spaces through contrast learning to achieve cross-modal alignment

BLIP [25]: Adopts the encoder-decoder architecture combined with data bootstrap technology to unify visual language understanding and generation capabilities

MIMN [13]: Includes two interactive memory networks to supervise textual and visual information with a given aspect, capturing both cross-modal and self-influences in the data.

ESAFN [26]: Integrates textual and visual information by leveraging attention mechanisms to capture intra-modal and inter-modal dynamics.

MTVAF [6]: Propose a multi-granularity visual-textual feature fusion model, convert images into image captions, facial descriptions, and optical characters, to align visual and text modalities, and enable effective cross-modal fusion.

Res-BERT+BL [1]: Connects the visual features extracted by ResNet with the hidden representation of BERT, and stacks another BERT Layer on top of it.

EF-CapTrBert [8]: Translates images into text and combines them with text inputs in the BERT encoder for self-attention fusion.

TomBERT [1]: Introduces a target attention mechanism inspired by self-attention to perform target-image matching for deriving target-sensitive visual representations, aiming to identify sentiment polarities over opinion targets.

HIMT [4]: Integrates semantic concepts from images, modeling aspect-text and aspect-image interactions hierarchically, and reducing the semantic gap between text and image representations.

HF-EKCL [27]: Generates captions for images to supplement text and visual features, utilizing a cross-attention mechanism and graph neural networks to capture interactions between modalities. It also applies contrastive learning to deepen the model’s understanding of sentiment features.

KEF-TomBERT [5]: Enhancing the visual attention and sentiment prediction capabilities of the ABMSA task by utilizing adjective–noun pairs extracted from images.

AMIFN [3]: Introduces aspect-guided textual and visual representations while leveraging coarse-grained sentence-image interactions and syntactic dependencies for graph-based aspect-guided text-image fusion, and can also dynamically filter noise.

MG-VTFM [7]: Integrates multi-granularity semantic, syntactic, and visual features for deep sentiment interactions. It employs a hierarchical graph attention network and progressive multimodal attention fusion to reduce noise, enhance sentiment relevance, and align cross-modal information based on aspect words.

### 4.4. Comparison with Baselines

Table 4 compares TESAM with existing methods on two ABMSA task datasets, focusing on accuracy and Macro-F1 scores. TESAM outperforms most models across metrics. On the TWITTER-17 dataset, TESAM improves accuracy by 0.08% and Macro-F1 by 0.89% compared to MG-VTFM. This improvement stems from TESAM’s integration of text-in-image information, enhancing visual representations, especially on datasets like TWITTER-17, where over 60% of images contain text. TESAM also handles noise effectively, such as in images consisting solely of text, which traditional models often overlook. On TWITTER-15, where only half of the images contain text, TESAM’s accuracy is 0.31% lower than MG-VTFM, but its F1 score improves by 1.21%. This suggests TESAM’s superior ability to manage noisy data by leveraging self-supervised learning for modal alignment, and TESAM reduces noise during feature fusion. To align different modalities, KEF-TomBERT and HF-EKCL convert images into text descriptions, which can lead to the loss of fine-grained visual information. As a result, their accuracy and F1 scores are slightly lower than those of TESAM, which retains visual features. TESAM’s F1 score on Multi-ZOL is 2.44% higher than that of HIMT, demonstrating its strong multi-scenario generalization capability, thus exhibiting equally outstanding performance in sentiment analysis of product reviews. TomBERT and EF-CapTrBert, while BERT-based, exhibit mixed performance. TomBERT’s approach of concatenating image and text features often introduces noise, affecting its performance on TWITTER-15. EF-CapTrBERT relies on converting images into textual descriptions, which enhances its performance on images with rich visual cues. This advantage enables its superior performance over TomBERT on Multi-ZOL, as the dataset contains substantial explicit complementarity between images and text (e.g., smartphone photos paired with product reviews). Res-BERT+BL offers a simpler fusion method, resulting in weaker performance compared to TomBERT and EF-CapTrBert. Although MTVAF uses text-in-image like TESAM, it relies on basic concatenation of image descriptions and text. In contrast, TESAM uses parallel cross-modal attention for bidirectional interaction between enhanced image representation (EIR) and text features, providing a comprehensive and accurate multimodal sentiment representation. This enables TESAM to achieve superior sentiment analysis performance. CLIP and BLIP exhibit moderate performance in the ABMSA tasks. As general models, their pre-training objectives prioritize global cross-modal alignment. As a result, they lack explicit aspect-word modeling and the ability to capture the fine-grained semantic and sentiment relationships required for ABMSA.

Moreover, in Table 5, we provide a comparison of different models’ trainable parameter counts. The table shows that TESAM has the fewest parameters and inference memory, the fastest inference speed, and achieves the highest scores. These results demonstrate the advantages of TESAM’s alignment methodology in reducing model complexity. By leveraging image captioning datasets for pre-training, TESAM eliminates the need for additional image understanding models to assist with cross-modal alignment, thereby reducing parameter count (164 M) and increasing inference speed (733.12 FPS). Furthermore, through parameter sharing mechanisms, TESAM achieves memory consumption under 1 GB during inference. As a result, TESAM achieves a balance between computational efficiency and robust performance in the ABMSA task, especially suitable for edge devices (e.g., smartphones and Raspberry Pi) with memory less than 4 GB, and uses limited power consumption for high-speed inference.

### 4.5. Ablation Study

#### 4.5.1. Impact of Proposed Methods

To demonstrate the effectiveness of each improvement, we conducted ablation experiments and present the results in Table 6.

Specifically, after masking the text-in-image, the model’s performance declined the most, with a decrease of approximately 3% in accuracy and 4% in F1 score. In this case, the model relies solely on visual cues from the image modality, ignoring the guidance provided by the embedded text, which results in a greater discrepancy between the model’s understanding and that of humans for multimodal samples. This indicates that images with embedded text are meaningful for accurate sentiment analysis in social media contexts, further validating the value of our innovative incorporation of text-in-image. Then, by incorporating aspect words into text-in-image within the IE, the model’s accuracy increased by approximately 1.2%, and the F1 score improved by about 1%. This demonstrates that aspect words play a crucial role in the ABMSA task. By guiding the model to focus on information relevant to the aspect words, we can reduce noise interference, thereby increasing the proportion of relevant information in higher-level sentiment features, ultimately leading to more accurate sentiment analysis. Next, skipping modal alignment with the image captions dataset and proceeding directly to the next training step results in a noticeable drop in accuracy and F1 score. This is because failing to align modal representations beforehand not only increases the model’s training complexity but also leads to weaker cross-modal interactions, especially for samples with ambiguous or noisy data. Finally, by disrupting the bidirectional interaction structure in the multimodal encoder (by replacing cross-modal attention with intra-modal self-attention), the model’s accuracy and F1 score drop by 2.67% and 3.21% on TWITTER-17. This is because ABMSA heavily relies on the comparative representation of aspects across different modalities. Thus, without cross-modal interaction, the model fails to capture deeply integrated sentiment features, leading to a noticeable decrease in performance.

The results show that omitting any of the improvements led to a decline in model effectiveness.

#### 4.5.2. Impact of Different OCR Methods

We compared the performance impact of different OCR tools: Tesseract [28], an open-source OCR engine based on LSTM, performs well on clear and well-formatted text. PaddleOCR [29], an OCR tool built on the PaddlePaddle framework, excels in handling complex images. MuggleOCR [30], a lightweight open-source OCR tool, is suitable for processing printed text and parsing captchas. EasyOCR [22], implemented with PyTorch, utilizes the CRAFT algorithm for character detection and CRNN for recognition, enabling it to handle various fonts, styles, and complex scenarios. The results are listed in Table 7.

As shown in the table, TESAM with EasyOCR achieved the best results on the TWITTER-15 dataset. On TWITTER-17, TESAM with PaddleOCR obtained an F1 score 0.07% higher than the former but had an accuracy 0.16% lower. This is because PaddleOCR has better adaptability to complex scenes, leading to improved class balance. Both Tesseract and MuggleOCR are designed for printed and clear text, making them more susceptible to noise when processing social media images, which negatively impacts subsequent sentiment analysis. Consequently, EasyOCR’s robustness in handling complex layouts and fonts enabled TESAM with EasyOCR to achieve the highest accuracy on both social media datasets. Additionally, since both EasyOCR and TESAM are implemented with PyTorch, their compatibility is enhanced. Therefore, we chose EasyOCR as the text recognition engine for TESAM.

#### 4.5.3. Impact of Different Hyperparameters

The purpose of this experiment is to provide a basis for the selection of hyperparameters.

**Figure 3:**
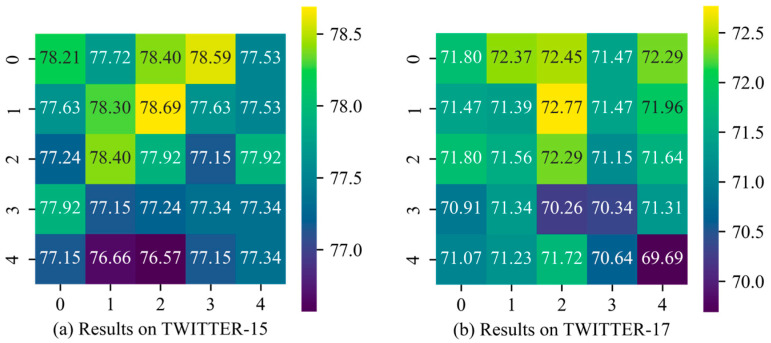
Heatmap of accuracy of hyperparameter selection results.

**Figure 4:**
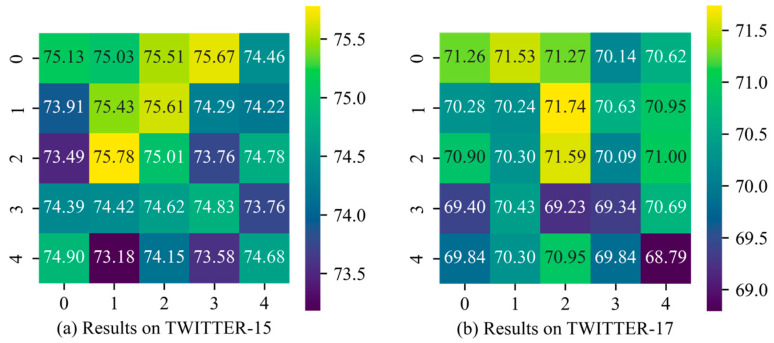
Heatmap of Macro-F1 of hyperparameter selection results.

We conducted experiments on the impact of hyperparameters *t* (the number of transformer encoder layers in the IE module) and *s* (the number of stacked layers in the BI module) on Twitter-15 and Twitter-17 datasets, and the results are shown in Figure 3 and Figure 4. The vertical axis represents *t*, while the horizontal axis represents *s*. The numbers within the grid indicate the model’s accuracy on the corresponding dataset under each hyperparameter configuration. The grid colors range from yellow to purple, with yellow representing higher accuracy and purple indicating lower accuracy.

For parameters *t* and *s*, we experimented with five values: {0, 1, 2, 3, 4}. Specifically, a value of 0 indicates that the TE layer is excluded from the IE or that the BI module has been removed. From Figure 4, it can be observed that when *t* = 1 and *s* = 2, TESAM achieves optimal performance on TWITTER-15 (accuracy: 78.69%), TWITTER-17 (accuracy: 72.77%), and F1 score (71.74%). However, as the value of *t* and *s* increases, the accuracy decreases. This indicates that increasing the number of internal layers in both encoders tends to make model training more challenging. Specifically, for the IE, when *t* > 1, the model accuracy starts to decline. On TWITTER-15, the highest accuracy at *t* = 4 is 0.38% lower than the lowest accuracy when the TE layer is absent from the IE. For the BI module, stacking additional BI layers can influence accuracy to some extent. While two BI layers capture more comprehensive cross-modal interactions compared to a single layer, when *s* > 2, the added parameters lead to increased training difficulty, causing a downward trend in model performance.

Additionally, when *t* = 2 and s = 1, TESAM reaches its peak F1 score on TWITTER-15 (75.78%). This phenomenon occurs because the stochastic gradient descent of model parameters causes performance metrics to randomly settle near local optima. Overall, the close alignment between accuracy and F1 score distributions demonstrates that variations in *t* and *s* parameters do not significantly affect the class balance (as measured by F1 score) in the model’s outputs.

### 4.6. Visualization and Case Study

To highlight the performance improvement of TESAM, we present several case studies in Table 8. For images with rich visual cues (e.g., Table 8(c)), TESAM’s bidirectional interaction structure captures relevant areas linked to aspect words and sentiment. For text-heavy images (e.g., Table 8(a)), the model extracts meaningful sentiment features from limited visual information, improving classification performance in fine-grained ABMSA tasks. The table includes aspect-level text (in parentheses) and its label (italicized and color-coded) for context. The “Text Attention” and “Text-in-image Attention” rows display the top five words most relevant to the aspect words, drawn from text and text-in-image, respectively. Lastly, the table compares predictions from different models on the same sample.

In case (a), the image feature extraction module with text recognition proposed in TESAM successfully captures key sentiment-related words in the image, such as “regret” and “sorry”, as shown in the Text-in-image Attention row. This enables TESAM to comprehensively extract sentiment features from the sample and establish relationships between features from different modalities, ultimately leading to accurate sentiment polarity classification of the aspect. In contrast, both AMIFN and MG-VTFM incorrectly classified “Roger Federer” as neutral. This error arises because the sentiment inclination of the text is not clearly evident, and both models overlook the text-in-image, missing crucial information in the image itself.

In case (b), AMIFN predicts a positive sentiment for “# Knoxville”, likely due to its use of a dynamic noise-filtering approach. Elements in the image, such as smiling faces and signs, indicate support for a wage increase, which may lead AMIFN to amplify positive sentiment. TESAM, on the other hand, accurately predicts a *neutral* sentiment for the aspect words. This is due to our approach of inserting aspect words within the text-in-image, guiding the model to focus on image regions related to the specific aspect while ignoring irrelevant features. As seen in the visual attention and Text-in-image Attention results, the model disregards elements that convey positive sentiment, such as faces. This results in a neutral sentiment analysis for the aspect term, demonstrating that our proposed method facilitates more accurate predictions by avoiding the influence of irrelevant sentiment features when the aspect itself is relatively independent.

In case (c), the three models under comparison produced different results, but only our TESAM provided the correct prediction. This accuracy is attributed to TESAM’s integration of text-in-image for the image modality, which enriches image representations by incorporating not only pure visual features (such as facial expressions) but also additional textual semantics that convey sentiment (e.g., “vote for Labour”). This addition enhances the model’s understanding of the sample. Furthermore, during the pre-training stage, TESAM’s self-supervised modal alignment ensures that the feature extraction module maps relevant semantics to relevant feature representations. This facilitates the BI module in effectively capturing the aspect target’s sentiment expressions across modalities. For instance, TESAM is able to interpret facial expressions in an image as strong support for “J. K. Rowling”, resulting in a correct positive classification.

## 5. Discussion

Although TESAM has achieved promising results on all three ABMSA datasets, and the effectiveness of the proposed method has been validated, there remain several issues that warrant further discussion.

### 5.1. Error Analysis of TESAM

To further refine our understanding of TESAM’s limitations, we analyze misclassified samples to delineate its applicability and identify potential directions for future research. Specifically, these errors can be categorized into three distinct types.

(1) The sample contains rhetorical questions or sarcasm. For example, in Table 9(a), the author poses a question, but TESAM incorrectly associates the aspect-level target with a smiling face in the image. Additionally, it misinterprets the phrase “will win” as a positive sentiment toward “Donald Trump”, failing to recognize the intended skeptical or ironic tone.
(2) The sample lacks explicit sentiment clues. As shown in Table 9(b), there is no obvious sentiment component related to ”The Zone” in both text and image, so TESAM chooses to classify it as neutral. However, combined with common sense, “The Zone” may be a radio channel, and the author is expressing his expectation for this activity and recommending this channel.
(3) Some of the aspects are too emotional when the sample contains multiple aspects. For example, in Table 9(c), the text is full of negative emotions about ”officialpepe”, when the fallen person and the other players in the image also express negative sentiment. This causes TESAM’s cross-modal attention to be overwhelmed by negative sentiment features, and makes the same judgment on other irrelevant aspects.

To address these issues, we plan to introduce methods such as sarcasm detection, knowledge graphs, and syntactic parsing in future work. These approaches will effectively enhance the model’s capabilities in handling abnormal tones and implicit sentiment expressions, while also equipping it with grammatical understanding to disentangle irrelevant aspect targets, ultimately improving the model’s sentiment analysis performance.

### 5.2. Potential Deviations in the Datasets

In our experiments, we utilized three types of datasets. The first is the image captioning dataset COCO-Captions, used for modal alignment. The second is the ABMSA task dataset TWITTER-15/17 from the social media domain, and the third is the ABMSA task dataset Multi-ZOL from the product review domain. These datasets may contain potential biases that could affect model performance or cause the model’s judgments to deviate from widely accepted values.

First, both COCO-Captions and TWITTER-15/17 are sourced from the Western-dominated internet. As a result, most images in these datasets feature people and scenes from the Western world, lacking representation of diverse cultures. This may lead to performance biases in cross-cultural scenarios. For example, in some Latin American cultures, a “funeral” is not considered a sorrowful event; instead, it may be understood as a cause for celebration. Therefore, it is necessary to expand existing benchmark datasets to include more diverse cultures. Thus, techniques such as knowledge graphs can be introduced to help models correctly handle the cultural and contextual differences that objectively exist in reality, leveraging rich prior knowledge.

Second, Multi-ZOL, as a Chinese smartphone review dataset, is limited to the single category of “smartphones”, and the brands covered are predominantly domestic Chinese brands. This results in the model learning features that are highly domain-specific, making it difficult to transfer to sentiment analysis tasks for other product reviews. It also introduces brand bias, failing to represent a broader user base. Therefore, in future work, we will incorporate more diverse cross-domain multimodal data to enhance the model’s generalization capabilities. Also, we plan to employ debiasing algorithms or domain adaptation methods to mitigate biases caused by dataset limitations, thereby improving TESAM’s applicability across different product categories and cultural contexts.

## 6. Conclusions

In this study, we propose the text-in-image enhanced self-supervised alignment model (TESAM) for aspect-based multimodal sentiment analysis (ABMSA), aiming to address the interference caused by images containing text in social media contexts and improve the representational differences between modalities without introducing additional parameters. TESAM introduces an image encoder capable of extracting text-in-image and leveraging it to enhance visual features. By inserting aspect words into the identified text-in-image, the image encoder guides TESAM to focus on regions of the image relevant to the aspect, thereby mitigating noise interference. Furthermore, TESAM leverages self-supervised alignment using the image caption dataset to bridge the semantic gap between modalities, effectively reducing adverse effects and ultimately enhancing ABMSA task performance. In comparative experiments, TESAM demonstrated superior performance in two benchmark ABMSA tests, achieving Macro-F1 values of 75.61% on the TWITTER-15 dataset, 71.74% on the TWITTER17 dataset, and 69.02% on the Multi-ZOL dataset, which is higher than traditional models. Moreover, subsequent ablation and case studies provide compelling evidence supporting the effectiveness of the proposed innovations.

Although our model demonstrates performance improvements, several limitations should be acknowledged. First, TESAM struggles to adapt to samples with rhetorical questions or those containing too little/too much emotional content. Second, the datasets used may contain potential contextual and cultural biases, which could lead to limitations in the model’s cross-cultural generalization capabilities. Third, in an effort to reduce model parameters, we did not treat aspect-level targets as independent textual inputs, unlike approaches such as TomBERT. This decision may have impacted the classification results. Additionally, the loss function used by TESAM for self-supervised alignment only measures feature distance. Therefore, further refining TESAM to ensure its strong performance across a broader range of MSA tasks is crucial. In our future work, we will incorporate methods such as syntactic parsing and knowledge graphs to enhance the model’s adaptability to special contexts and cross-cultural scenarios. We will also introduce distribution alignment techniques to improve cross-modal feature distribution alignment, as well as diversify training samples or employ domain adaptation methods to account for the impact of different domains or cultural elements on sentiment analysis results.

## Figures and Tables

**Table 1:** Examples of input tokens with aspect inserted.

Aspect	Example
Gato	[CLS] Gato [SEP] Jeremy’s cat Gato just threw some glorious shade at David [SEP]
Calum	[CLS] Calum [SEP] Calum is me on the first day of school #ShesKindaHotVMA [SEP]

**Table 2:** Basic statistics of TWITTER datasets.

Dataset		Negative	Neutral	Positive	Total	Text-Containing Image
TWITTER-15	Train	368	1883	928	3179	51.05%
	Dev	149	670	303	1122	53.21%
	Test	113	607	317	1037	53.04%
TWITTER-17	Train	416	1638	1508	3562	62.58%
	Dev	144	517	515	1176	66.33%
	Test	168	573	493	1234	61.59%

**Table 3:** Basic statistics of Multi-ZOL dataset.

Labels	Train	Dev	Test	Total	Text-Containing Image
1, 2, 3, 4 (negative)	1152	148	139	1439	65.25%
5, 6, 7 (neutral)	4670	588	581	5839	
8, 9, 10 (positive)	16,921	2107	2123	21,151	

**Table 4:** Comparison with baselines on ABMSA task. The bold indicates the optimal values.

Method	Dataset					
	TWITTER-15		TWITTER-17		Multi-ZOL	
	Acc/%	F1/%	Acc/%	F1/%	Acc/%	F1/%
CLIP	67.40	45.27	57.29	51.63	-	-
BLIP	73.87	68.53	67.91	64.39	-	-
MIMN	71.84	65.69	65.88	62.99	61.59	60.51
ESAFN	73.38	67.37	67.83	64.22	-	-
MTVAF	-	71.00	-	68.20	-	-
Res-BERT+BL	75.02	69.21	69.20	66.48	65.19	64.98
EF-CapTrBert	78.01	73.25	69.77	68.42	68.20	67.55
TomBERT	77.15	71.75	70.50	68.04	65.04	64.71
HIMT	78.14	73.68	71.14	68.16	66.83	66.58
HF-EKCL	78.38	75.39	71.37	69.88	-	-
KEF-TomBERT	78.68	73.15	72.12	69.69	-	-
AMIFN	78.69	75.50	72.29	70.21	-	-
MG-VTFM	**79.00**	74.40	72.69	70.85	-	-
TESAM (ours)	78.69	**75.61**	**72.77**	**71.74**	**69.33**	**69.02**

**Table 5:** Comparison of model’s trainable parameter count and inference speed. The bold indicates the optimal values.

Method	Parameter Count	Inference Speed/FPS	Inference Memory/GB	TWITTER-17		Multi-ZOL	
				Acc/%	F1/%	Acc/%	F1/%
TomBERT	235 M	548.48	1.2	70.50	68.04	65.04	64.71
HIMT	303 M	522.88	1.5	71.14	68.16	66.83	66.58
MG-VTFM	270 M	-	-	72.69	70.85	-	-
TESAM (ours)	**164 M**	**733.12**	**0.8**	**72.77**	**71.74**	**69.33**	**69.02**

**Table 6:** Results of TESAM w/o proposed methods. The bold indicates the optimal values.

Method	Dataset			
	TWITTER-15		TWITTER-17	
	Acc/%	F1/%	Acc/%	F1/%
w/o Text-in-image	75.51	71.74	69.44	67.41
w/o Aspect Insert	77.53	73.28	71.47	70.99
w/o Alignment	76.95	73.10	70.75	68.46
w/o MCA	75.89	71.28	70.10	68.53
TESAM (ours)	**78.69**	**75.61**	**72.77**	**71.74**

**Table 7:** Results of TESAM with different OCR tools. The bold indicates the optimal values.

Method	Dataset			
	TWITTER-15		TWITTER-17	
	Acc/%	F1/%	Acc/%	F1/%
with Tesseract	78.21	74.86	72.37	71.16
with PaddleOCR	78.50	75.51	72.61	**71.81**
with MuggleOCR	77.05	74.67	71.96	70.04
with EasyOCR	**78.69**	**75.61**	**72.77**	71.74

**Table 8:** Visualization results and right cases. The colored overlays in the figure show the output weights from the first BI layer. Red regions represent areas of interest, while blue regions indicate less relevant areas. “×”indicates incorrect predictions, while “√”denotes correct predictions.

Visual Attention	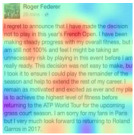	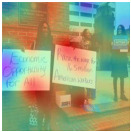	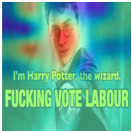
Text	(a) Statement from [Roger Federer]_negative_ regarding his withdrawal from Roland Garros.	(b) RT @ OFA TN: These ladies in [# Knoxville]_neutral_ know # RaiseTheWage creates better economic opportunity for all Americans	(c) Right. If [J. K. Rowling]_positive_ won’t post this then I will:
Text Attention	from, Roger, withdrawal, his, Roland	for, creates, know, of, all	I, will, if, j, k
Text-in-image Attention	have, made, regret, sorry, decision	[SEP], [SEP], workers, [PAD], for	Harry, wizard, [SEP], vote, Potter
AMIFN	(neutral ×)	(positive ×)	(neutral ×)
MG-VTFM	(neutral ×)	(neutral √)	(negative ×)
TESAM	(negative √)	(neutral √)	(positive √)

**Table 9:** Misclassified samples by TESAM. “×”indicates incorrect predictions, while “√”denotes correct predictions.

Image	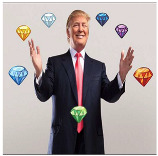	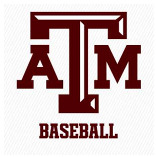	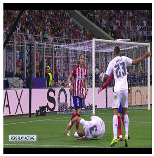
Text	(a) Meta Theory: [Donald Trump]_neutral_ will win the election WITH THE POWER OF THE CHAOS EMERALDS?!	(b) Listen to @ [AggieBaseball]_positive_ take on [TCU]_negative_ in the elimination game tomorrow at 12:45 p.m. on [The Zone]_positive_!	(c) This is @ [officialpepe]_negative_. He is a disgrace. He is pathetic. And he is an embarrassment to football. @ [realmadriden]_neutral_
TESAM’s prediction	(positive ×)	(positive √, negative √, neutral ×)	(negative √, negative ×)

## Data Availability

The datasets used in this study are publicly available. TWITTER-15 and TWITTER-17 datasets can be accessed via: https://github.com/jefferyYu/TomBERT (13 April 2025). The Multi-ZOL dataset can be accessed via: https://github.com/xunan0812/MIMN (13 April 2025). COCO-Captions can be accessed via: https://cocodataset.org/#download (13 April 2025).
